# B-vitamin consumption and the prevalence of diabetes and obesity among the US adults: population based ecological study

**DOI:** 10.1186/1471-2458-10-746

**Published:** 2010-12-02

**Authors:** Shi-Sheng Zhou, Da Li, Yi-Ming Zhou, Wu-Ping Sun, Qi-Gui Liu

**Affiliations:** 1 Institute of Basic Medical Sciences, Medical College, Dalian University, Dalian 116622, PR China; 2 Okazaki Institute for Integrative Bioscience, National Institutes of Natural Sciences, Okazaki 444-8787, Japan; 3 Department of Health Statistics, Dalian Medical University, Dalian 116044, PR China

## Abstract

**Background:**

The global increased prevalence of obesity and diabetes occurred after the worldwide spread of B-vitamins fortification, in which whether long-term exposure to high level of B vitamins plays a role is unknown. Our aim was to examine the relationships between B-vitamins consumption and the obesity and diabetes prevalence.

**Methods:**

This population based ecological study was conducted to examine possible associations between the consumption of the B vitamins and macronutrients and the obesity and diabetes prevalence in the US population using the per capita consumption data from the US Economic Research Service and the prevalence data from the US Centers for Disease Control and Prevention.

**Results:**

The prevalences of diabetes and adult obesity were highly correlated with per capita consumption of niacin, thiamin and riboflavin with a 26-and 10-year lag, respectively (*R*^2 ^= 0.952, 0.917 and 0.83 for diabetes, respectively, and *R*^2 ^= 0.964, 0.975 and 0.935 for obesity, respectively). The diabetes prevalence increased with the obesity prevalence with a 16-year lag (*R*^2 ^= 0.975). The relationships between the diabetes or obesity prevalence and per capita niacin consumption were similar both in different age groups and in male and female populations. The prevalence of adult obesity and diabetes was highly correlated with the grain contribution to niacin (*R*^2 ^= 0.925 and 0.901, respectively), with a 10-and 26-year lag, respectively. The prevalence of obesity in US adults during 1971-2004 increased in parallel with the increase in carbohydrate consumption with a 10-year lag. The per capita energy and protein consumptions positively correlated with the obesity prevalence with a one-year lag. Moreover, there was an 11-year lag relationship between per capita energy and protein consumption and the consumption of niacin, thiamin and riboflavin (*R*^2 ^= 0.932, 0.923 and 0.849 for energy, respectively, and *R*^2 ^= 0.922, 0.878 and 0.787 for protein, respectively).

**Conclusions:**

Long-term exposure to high level of the B vitamins may be involved in the increased prevalence of obesity and diabetes in the US in the past 50 years. The possible roles of B-vitamins fortification and excess niacin consumption in the increased prevalence of obesity and diabetes were discussed.

## Background

Obesity and type 2 diabetes have been a major global health problem [1,2]. It is during the past few decades that obesity and type 2 diabetes have rapidly reached epidemic proportions not only in adults but also in children and adolescents [3,4]. The fact that human genome has not changed markedly in such a short time has led to the hypothesis that obesity and diabetes are the result of gene-environment/diet interaction [5,6]. Increasing evidence has indicated that diet factors may play a crucial role in promoting obesity and type 2 diabetes [6,7]. Recently, considerable attention has been focused on dietary carbohydrates, for the epidemiological studies from the United States (US) have shown that the rising prevalence of obesity and type 2 diabetes has been accompanied by a significant increase in carbohydrate consumption during the past three decades [8-10]. However, the nature of risk factors in carbohydrate diets remains unclear [11,12].

Since around the mid-20th century, one of the major changes in diet has been the significant increase in the content of niacin (vitamin B_3_, either nicotinic acid or nicotinamide), thiamin and riboflavin in grain (including flour) products, because of the worldwide spread of B-vitamins fortification, i.e., the addition of the B vitamins to a food above the level normally to prevent the deficiency of the vitamins [13]. One likely possibility is that long-term intake of B-vitamins-fortified foods may lead to a chronic overload of the B vitamins. For example, the amount of daily niacin consumption per capita in the US has increased from 16 mg in the late 1930s (just before the implementation of niacin fortification) to 33 mg in the early 2000s [14], a level of more than 2 times higher than the recommended dietary allowance (RDA) by the US Food and Nutrition Board (RDA: 14 and 16 mg/d for adult women and men, respectively) [15]. Obviously, a largely ignored fact is that the rapid increase in the global prevalence of obesity and type 2 diabetes has occurred following the worldwide spread of the B-vitamins fortification of foods. The overall trend is that the global increasing prevalence of obesity and type 2 diabetes occurred first in the earliest fortified-countries, and then spread to the latter fortified-countries. Whether the increased prevalence of obesity and type 2 diabetes in the past few decades involves excess consumption of the B vitamins is not known.

Moreover, with the implementation of B-vitamins fortification of grains, the major source of carbohydrates, the effect of high carbohydrate diets on obesity and diabetes has significantly changed in the past three decades. Before the introduction of B-vitamins fortification of grains, a high-carbohydrate dietary pattern had been known to be associated with a low prevalence of obesity and type 2 diabetes, and a low-fat, high-carbohydrate diet is the traditional recommendation for treating type 2 diabetes [16-18]. However, this traditional dietary recommendation has been challenged by the epidemiological evidence from the US that adopting high-carbohydrate, low-fat diets in the past three decades have been unexpectedly followed by a sudden sharp increase of obesity prevalence starting from the early 1980s. Although it is argued that high carbohydrate intake may increase the risk for obesity and type 2 diabetes [8-10], what underlies the change in the effect carbohydrate diets remains unanswered. It should be noted that the sudden sharp increase in the nationwide prevalence of obesity in the US has occurred soon after the update of B-vitamins fortification standards in 1974, which has led to a significant increase in the B-vitamins contents in grain products since then. However, the relationship between these two events remains to be investigated.

Among the three fortified B vitamins, niacin is well known to induce severe adverse effects, including glucose intolerance, insulin resistance and liver injury [15,19,20], all of which are the major hallmarks of obesity and type 2 diabetes [2,4]. Our previous study suggested that type 2 diabetes and children obesity may involve excess niacin intake [21,22]. Thus, a high prevalence of impaired glucose tolerance, insulin resistance, and subsequent obesity and type 2 diabetes is expected to occur in the population exposed to long-term high intake of the B vitamins. To test this possibility, this ecological study examined the associations between the prevalence of adult obesity and diabetes in the US and the per capita consumption of niacin, thiamin and riboflavin, and main macronutrients (carbohydrate, protein, saturated fat, dietary fiber) as well.

## Methods

### Data sources

This ecological study investigated the association between an exposure (B-vitamins consumption) and outcomes (the prevalence of obesity and diabetes) using aggregated data on a population level (the US population), and was conducted by analyzing obesity and diabetes prevalence data from all of the participants in the US National Health Interview Survey (NHIS), National Health Examination Survey (NHES) and National Health and Nutrition Examination Surveys (NHANES).

The data on the per capita nutrient and energy consumption in 1909-2004 [23], the per capita grain consumption in 1909-2007 [24], the grain contribution to niacin consumption and the energy consumption from major food groups in 1909-2000 [14] were derived from the databases of the Economic Research Service (ERS) of the US Department of Agriculture. ERS annually calculates the amounts of several hundred foods available for human consumption in the US and provides estimates of per capita availability. In brief, the food consumption (or food disappearance) is calculated at the national level by adding total annual production, imports, and beginning stocks of a particular commodity and then subtracting exports, ending stocks, and nonfood uses. Per capita estimates are calculated using population estimates for that particular year. To estimate grain contribution to total niacin, total niacin contributed from daily per capita consumption of a variety of food items, mainly including meat, poultry, fish, grain products, milk, cheese, legumes, fruits, vegetables, etc, is calculated according to the niacin content of each food, and then the amount of niacin from grains was divided by the total niacin amount (see Table nineteen in Ref. [14]). ERS's food availability (per capita) data serve as indirect measures of trends in food use. The Food Availability (Per Capita) Data System provides an indication of whether Americans, on average, are consuming more or less of various foods over time. Also, the estimates of nutrients in the food supply reflect Federal enrichment and fortification standards and technological advances in the food industry [14].

The prevalence of diagnosed diabetes in the US population in 1958-2008 and in the all age groups of 0-44, 45-64, and 65-74 years of both sexes in the US population in 1980-2006 were derived from the NHIS of the National Center for Health Statistics (NCHS), Centers for Disease Control and Prevention (CDC) [25-27]. Conducted continuously since 1957, the NHIS is a health survey of the civilian, noninstitutionalized population of the US. The survey provides information on the health of the US population, including information on the prevalence and incidence of disease. The multistage probability design of the survey has been described elsewhere [28,29]. During 1980-1996 NHIS, each year, a one-sixth sub-sample of NHIS respondents was asked whether in the past 12 months they or any family member had diabetes. Three-year averages were used to improve the precision of the annual estimates. The NHIS was redesigned in 1997. In the redesigned survey, all sampled adults are asked whether a health professional had ever told them they had diabetes. To exclude gestational diabetes, women were asked whether they had been told they had diabetes other than during pregnancy. Also, parents of sampled children were asked whether their child had diabetes. Diabetes prevalence estimates are presented by age, race, ethnicity, and sex. Prevalence estimates were age-adjusted using NCHS estimates of the 2000 US population as the standard. Detailed descriptions of the survey methods are available on-line [26,27].

The data on the prevalence of obesity (body mass index ≥30.0) in adults aged 20-74 years in the US of both sexes were derived from the CDC's NHES (1960-1962), NHANES I (1971-1974), NHANES II (1976-1980), NHANES III (1988-1994), and the continuous NHANES 1999-2000, 2001-2002, 2003-2004 [30]. NHANES includes a series of cross-sectional nationally representative health examination surveys beginning in 1960. Beginning in 1999, NHANES became a continuous survey without a break between cycles. Each cross-sectional survey provides a national estimate for the US population at the time of the survey, enabling examination of trends over time. The survey examines a nationally representative sample of about 5,000 persons each year. The participants are located in counties across the country, 15 of which are visited each year. All participants visit the physician. Dietary interviews and body measurements are included for everyone. Health interviews are conducted in respondents' homes. Health measurements are performed in specially-designed and equipped mobile centers, which travel to locations throughout the country. The study team consists of a physician, medical and health technicians, as well as dietary and health interviewers. Detailed descriptions of the survey methods are available elsewhere [31,32], and on-line http://www.cdc.gov/nchs/nhanes/about_nhanes.htm.

### Statistical analyses

A time-lag regression analysis for the prevalence of diabetes and adult obesity as a function of per capita nutrient intake was carried out to determine if per capita nutrient intake had a time-delayed effect on the prevalence of diabetes and obesity. Using SPSS software (SPSS Inc., Chicago, USA), each lag regression analysis was performed with an initial lag value of zero, and then the lag value for changes in the prevalence of diabetes and adult obesity was increased by a step of one year until the maximum coefficient of determination (*R*^2^) was obtained. Thus the lag time between a given nutrient intake and the prevalence of diabetes or obesity was determined. Then, graph of the prevalence of diabetes and adult obesity against a given nutrient intake was plotted according to the lag time. A similar regression analysis was used to examine the possible relationship between intake changes of different nutrients. Statistical significance was set at *P *< 0.05. The data used for this study are available upon request.

## Results

### The per capita niacin consumption and the prevalence of diabetes in the US

**Figure 1:**
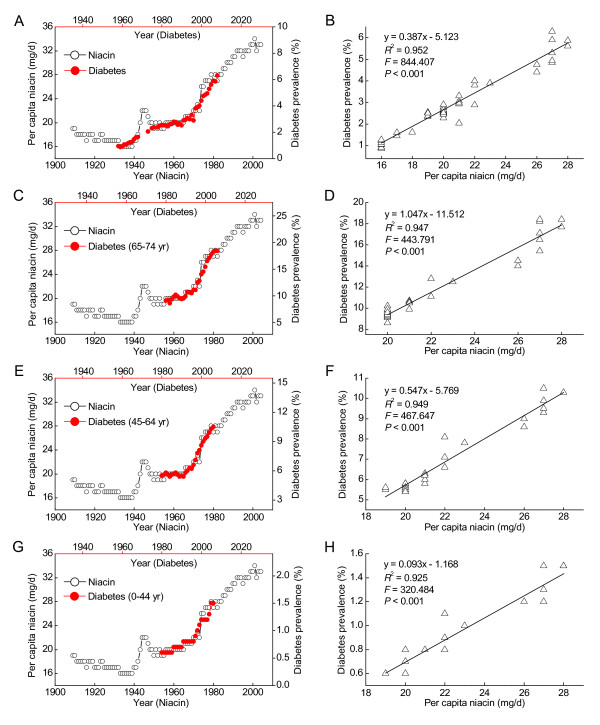
**The correlations between the per capita niacin consumption and the diabetes prevalence in the US**. A: The trends in the niacin consumption from 1909 to 2004 (Ref. [23]) and in the diabetes prevalence in the US in 1958-2008 (Ref. [25]). Note: the data on the prevalence of diabetes in 1969-1972, 1974 and 1977 are not available. B: The 26-year lag-regression plot using the data in A. C, E and G: The trends in the niacin consumption and in the diabetes prevalence in different age groups (Ref. [27]). D, F and H: The lag-regression plots using the data in C, E and G, respectively. The lag time is 24, 26 and 26 years for 65-74, 45-64, and 0-44 years age groups, respectively.

As shown in Figure 1 (open cycles), there were two sharp increasing periods in the per capita niacin consumption in the US: one started in 1938 and continued to the early 1940s, after which the daily per capita niacin consumption increased from 16 mg in 1939 to 20 mg in 1949; the other occurred since 1974, which has further increased the daily per capita niacin consumption from 22 mg in the early 1970s to 33 mg in the early 2000s [23]. Following the two sharp increases in the per capita niacin consumption, there were also two periods of rapid increase in the prevalence of diabetes in the US in the latter half of the 20th century: the first one started from the early 1960s to the mid-1970s, after which the prevalence abruptly increased from 0.87% in 1959 to 2.49% in 1979; the second one began in the mid-1990s. By 2008, the prevalence had increased from 2.52% in 1990 to 6.29%. Between the two periods, there was a relatively constant prevalence of diabetes. Most evidently, the prevalence of diabetes in the US population during 1958-2008 increased in striking parallel with the per capita niacin consumption in 1932-1982 (Figure 1A). Lag-regression analysis revealed that the prevalence of diabetes was determined by the per capita niacin consumption with a lag of 26 years (Figure 1B). The associations between the prevalence of diabetes in the US in 1980-2006 [26] and the per capita niacin consumption in 1954-1980 were similar in both sexes (*R*^2 ^= 0.958 and 0.935 for the male and female respectively, both *P <*0.001). Such a significant correlation was also found in each adult age group with a distinct lag time (24, 26 and 26 years lag for the age groups of 65-74, 45-64 and 0-44 years, respectively) (Figure 1C-H).

### The per capita grain consumption and the prevalence of diabetes in the US

**Figure 2:**
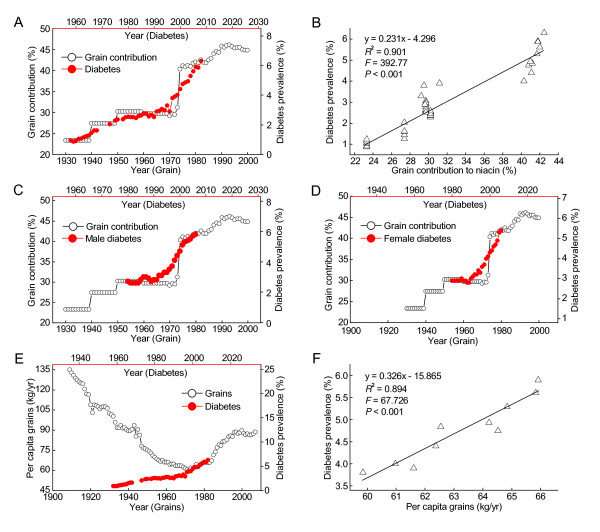
**The correlations between the grain contribution/consumption and the diabetes prevalence in the US**. A: The trends in the grain contribution to daily per capita niacin consumption (Ref. [14]) and in the diabetes prevalence (Ref. [25]). B: The 26-year lag-regression plot using the data in A. C and D: showing the similar relationships in both sexes. The diabetes data are from Ref. [26]. E: The trends in the per capita grain consumption (Ref. [24]) and the diabetes prevalence (Ref. [25]). F: The lag-regression plot of the diabetes prevalence in 1997-2008 against the grain consumption in 1971-1982 using the data in E.

Grains, the vehicle for niacin fortification, have become the major contributor to dietary niacin since the implementation of niacin fortification in the US. Niacin fortification has made the grain contribution to dietary niacin increase from 22.5% in 1930s (i.e., before niacin fortification) to 44.8% in 2000 [14]. As shown in Figure 2A, each of the two sharp increases in the grain contribution, occurred respectively in 1940s and in mid-1970s, was followed by a subsequent rapid increase in the prevalence of diabetes with a lag of 26 years. The prevalence of diabetes in the US in 1958-2008 was significantly correlated with the grain contribution to niacin in 1932-1982 (Figure 2B). The associations were similar not only in both sexes (Figure 2C and 2D, *R*^2 ^= 0.833 and 0.791 respectively for the male and female populations, both *P *< 0.001), but also in different age groups (*R*^2 ^= 0.801, 0.835 and 0.875 for the groups aged 0-44, 45-64 and 65-74 years, respectively, all *P <*0.001). Figure 2E shows that, in the early 20th century, the dietary pattern of high grain consumption was associated with a low prevalence of diabetes in the US. However, the re-increase in the consumption of niacin-fortified grains has been followed by a more rapid increase rather than decrease in the prevalence of diabetes in the US. The prevalence of diabetes in the US in 1997-2008 was significantly correlated with grain consumption in 1971-1982 (Figure 2F).

### The per capita niacin and grain consumption and the prevalence of obesity in US adults

**Figure 3:**
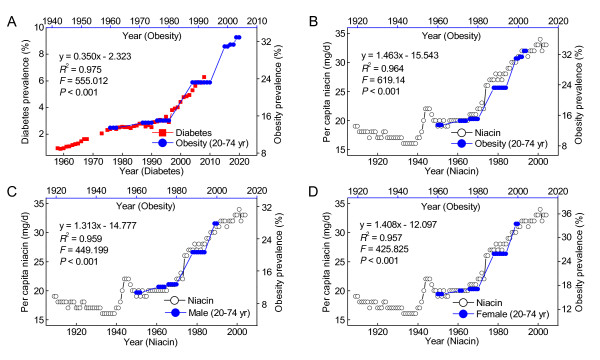
**The correlations between the niacin consumption and the obesity prevalence in US adults**. A: Trends of prevalence of obesity and diabetes in the US. B: The trends in the per capita niacin consumption (Ref. [23]) and in the obesity prevalence (Ref. [30]). The 10-year lag regression result is presented in the panel. C and D: showing the similar associations in both sexes. The obesity prevalence data are derived from Ref. [31]. The lag regression results are presented in each panel.

**Figure 4:**
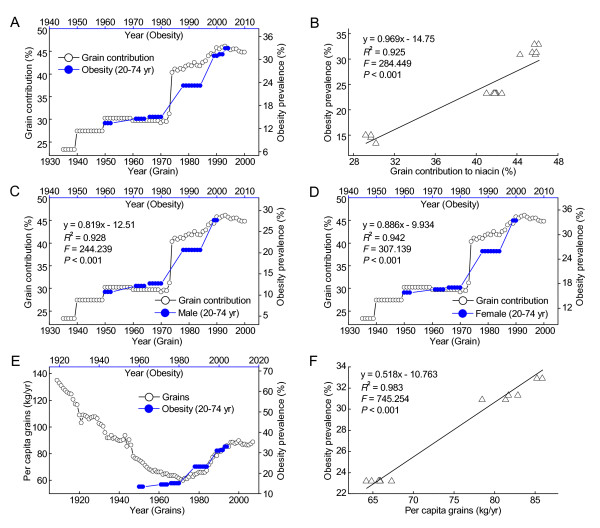
**The correlations between the grain contribution and consumption and the obesity prevalence in US adults**. A: The trends in the grain contribution to niacin (Ref. [14]) and the obesity prevalence (Ref. [30]). B: The 10-year lag-regression plot using the data in A. C and D show the similar correlations in both sexes. E: The trends in the yearly grain consumption (Ref. [24]) and the obesity prevalence (Ref. [30]). The 10-year lag regression result is presented in each panel. F: The lag-regression plot of the obesity prevalence in 1988-2004 against the per capita grain consumption in 1978-1994 using the data in E.

Obesity is a known risk factor for type 2 diabetes. The present results showed that the prevalence of diabetes in the US is significantly correlated with the prevalence of obesity with a time lag of 16 years (Figure 3A). The prevalence of obesity was significantly positively correlated with the daily per capita niacin consumption with a 10-year lag (Figure 3B). The correlations were similar in both sexes (Figure 3C and 3D), in the female age groups of 20-39 and 40-59 years (*R*^2 ^= 0.971 and 0.977, respectively, both *P *< 0.001), and in the male age groups of 20-39 years and 40-59 years (*R*^2 ^= 0.842 and 0.98, respectively, both *P *< 0.001). Figure 4 (A and B) shows that the prevalence of obesity among the US adult population in 1960-2004 increased in parallel with the increase in the grain contribution to niacin in 1950-1994. Similar relationships were observed in both sexes (Figure 4C and 4D). Moreover, as shown in Figure 4E, the re-increase in the consumption of niacin-fortified grains since the early 1970s has been followed by a more rapid increase in the prevalence of obesity in the US. The prevalence of obesity in the US adult population in 1988-2004 was significantly correlated with the per capita grain consumption in 1978-1994 (Figure 4F).

### The per capita thiamin and riboflavin consumption and the prevalence of obesity and diabetes in the US

**Figure 5:**
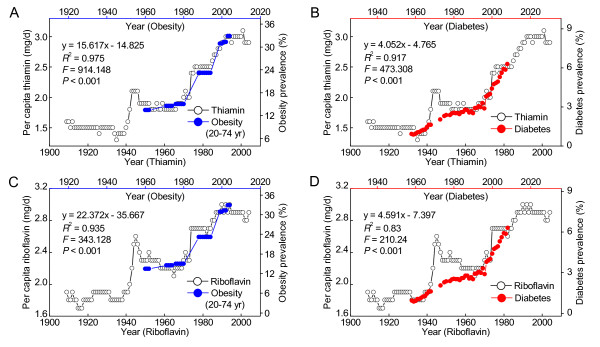
**Correlations between thiamin and riboflavin consumption and the prevalence of obesity and diabetes in the US**. A and B: The trends in the per capita thiamin consumption and the prevalence of obesity and diabetes. C and D: The trends in the per capita riboflavin consumption and the prevalence of obesity and diabetes. The 26-year lag regression results are presented in each panel.

Both thiamin and riboflavin, other B vitamins, have also been used to fortify grains in the US since the initiation of grain fortification in the early 1940s [14]. As shown in Figure 5 the implementation of mandatory fortification of grains had also led a rapid increase in per capita consumption of thiamin and riboflavin since the early 1940s, and the update of the fortification standards in 1974 led to a further sudden increase in the consumption of these two B vitamins (Figure 5A and 5C, open cycles). The present analysis revealed that the prevalence of obesity and diabetes increased in parallel with the increase in the consumption of thiamin and riboflavin, with a time lag of 10 and 26 years, respectively (Figure 5).

### The per capita macronutrient consumption and the prevalence of obesity and diabetes in the US

**Figure 6:**
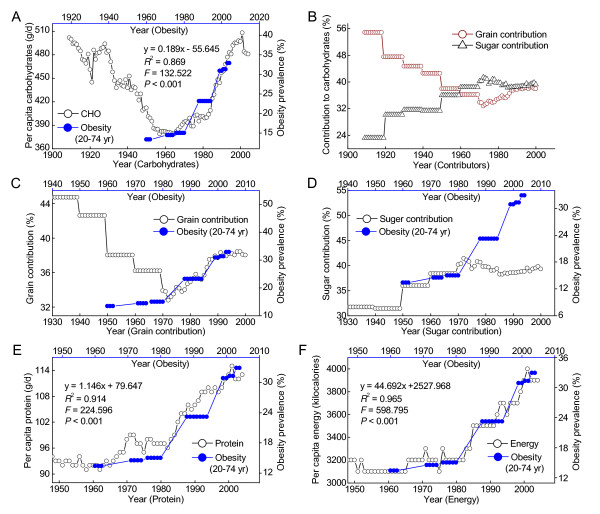
**Relationships between carbohydrate, protein and energy consumption and the obesity prevalence in the US**. A: The trends in the per capita carbohydrate consumption (Ref. [23]) and the obesity prevalence (Ref. [30]). Lag-regression result of the relationship between the per capita carbohydrate consumption in 1961-1994 and the obesity prevalence in 1971-2004 is given in the panel. CHO = carbohydrate. B: The trends in grain and sugar contribution to total carbohydrate (Ref. [14]). C and D: The relationships between grain and sugar contribution to daily per capita carbohydrate consumption and the obesity prevalence. E and F: respectively showing that the per capita protein and energy consumption increased in parallel with the obesity prevalence with a one-year lag. The one-year lag-regression result is presented in each panel.

**Figure 7:**
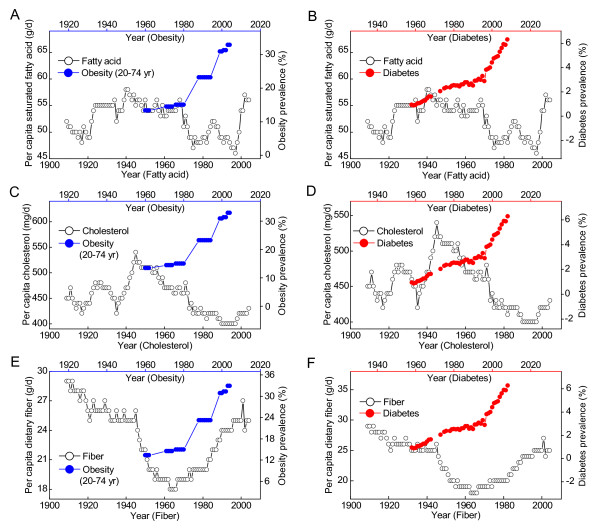
**The consumption of saturated fat, cholesterol and fiber and obesity and diabetes prevalence in the US**. A, C and E, The relationships between the obesity prevalence (Ref. [30]) and the per capita consumption of saturated fat, cholesterol and fiber (Ref. [23]). B, D and F, The relationships between the diabetes prevalence (Ref. [25]) and the per capita consumption of saturated fat, cholesterol and fiber.

Figure 6A shows that the trends in the per capita carbohydrate consumption and the obesity prevalence in the 20th century. Re-increase in carbohydrate consumption since the late 1960s was followed by a significantly increasing prevalence of obesity. Grain and sugar (including sweeteners) are two major contributors to dietary carbohydrate. Since the early 1970s, the per capita carbohydrate consumption has shown a trend of increase in the fortified-grain contribution and decrease in sugar contribution (Figure 6B). However, as shown in Figure 6C and 6D, this regimen did not prevent the increasing trend, but rather was followed by a steep increase in the prevalence of obesity. Protein is another important macronutrient. The per capita consumption of protein and energy has showed a significantly increasing trend since the late 1960s, and increased with the increase in the obesity prevalence with a one-year lag (Figure 6E and 6F, respectively). Moreover, there was a decreasing trend in US per capita consumption of dietary saturated fats in 1970s-1990s (Figure 7A, open cycles) and cholesterol from mid-1940s to late 1990s (Figure 7C, open cycles), with an increasing trend in the consumption of dietary fiber from mid-1960s to the early 2000 (Figure 7E, open cycles). Unexpectedly, all of these regimens have failed to prevent the increasing trends in the prevalence of obesity and diabetes in the last three decades of 20th century (Figure 7).

### Relationships between per capita energy consumption contributed from major food groups and the prevalence of adult obesity in the US

**Figure 8:**
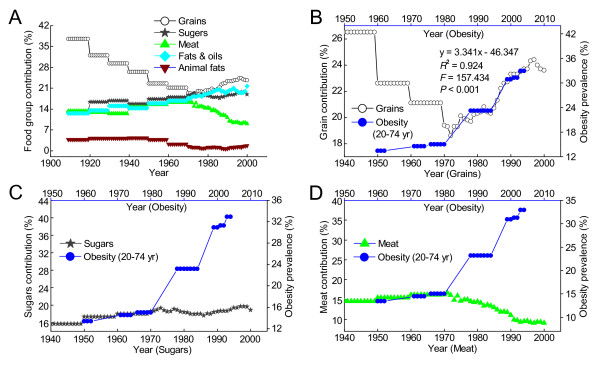
**Per capita energy consumption contributed from major food groups and the prevalence of obesity in US adults**. A: Contributions to energy consumption from grains, sugars, meat, fats/oils and animal fats (Ref. [14]). B-D: Relationships between the obesity prevalence and the contributions of grains, sugars and meat, respectively.

As shown in Figure 8A, most of the energy consumed by the US population is obtained from grains, sugars, meat and fats/oils. Among the contributions, the biggest is the grain contribution which has undergone a dramatic change in the last century. The data showed that high grain contribution to energy consumption was associated with very low obesity prevalence in the US population in the early 20th century. However, re-increase in the contribution of grains fortified with more B-vitamins since the early 1970s was followed by a sharp increase in the obesity prevalence. There is a significant correlation between the grain contribution to the energy consumption in 1969-1994 and the obesity prevalence in 1979-2004 (Figure 8B). In contrast, there were no significant positive correlations between the increased obesity prevalence and the contributions from other main energy contributors, including the known risk factors such as animal fats (Figure 8A), sugars (Figure 8C) and meat (Figure 8D).

### Relationships between per capita B-vitamins consumption and the consumption of energy and protein in the US

**Figure 9:**
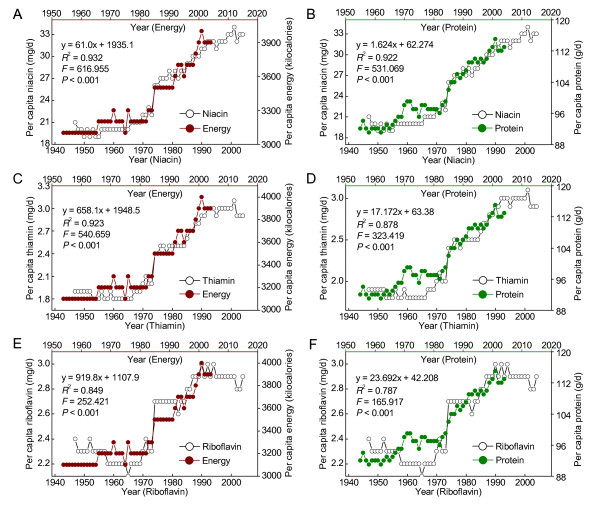
**Relationships between per capita B-vitamin consumption and energy and protein consumption in the US**. A-F: Correlations between the consumption niacin, thiamin and riboflavin and the consumption of energy and protein in the US. The data on per capita consumption of energy and the main nutrients were derived from the databases of the ERS of the US (Ref. [23]). The 11-year lag-regression results are presented in each panel.

As shown in Figure 9 there was a sudden increase in the consumption of both energy and protein in the US around mid-1980s, about 10 years after the update of the fortification standards, and since then, the per capita consumption of energy and protein has been showing an increasing upward trend. Lag-regression analysis revealed that there were significant correlations between the consumption of energy and protein and the consumption of niacin, thiamin or riboflavin with a time lag of 11 years, one year longer than that between the adult obesity prevalence and the per capita consumption of the B vitamins (see Figure 3B, 5A and 5C for comparison).

## Discussion

Obesity and type 2 diabetes are closely linked to diet. The increasing global prevalence of obesity and type 2 diabetes implies that there might have been some common, worldwide changes happened in diet. Indeed, a significant change, happened since around the mid-20th century, is food fortification with B-vitamins. The present ecological study found that the nationwide prevalence of obesity and diabetes in the US in the past 50 years increased in close parallel with the per capita consumption of niacin, thiamin or riboflavin, with a 10-and 26-year lag, respectively. It is obvious that the B-vitamins fortification has been followed first by an increase in the prevalence of obesity, and then by an increase in the prevalence of diabetes. Thus, it seems that the high level consumption of the B vitamins, primarily due to the mandatory grain fortification with the vitamins, may be an attractive candidate for the dietary changes responsible for the increased prevalence of obesity and diabetes.

### B-vitamins fortification of grains and the change in high-carbohydrate diet effect

The prevalence of diabetes began to rapidly increase in the US in the 1960s, which was thought to be possibly due to a shift towards a dietary pattern characterized by low fiber and high saturated fats and sugar. Since around the early 1970s, a series of preventive measures have been taken, including reducing consumption of saturated fats and sugar and increasing intake of grains and dietary fiber. Moreover, the standards of B-vitamins fortification were updated in 1974, which has led to a further significant increase in the B-vitamins contents in grain products [14]. Unexpectedly, all of these preventive measures have been followed by a sharp nationwide increase in the prevalence of obesity started from mid-1980s and a second rapid increase in the prevalence of diabetes in the late 1990s. Because one of the most significant changes during this period was the significant increase in the per capita grain consumption, it is suspected that there must be something happened in dietary carbohydrates [11].

Carbohydrates are the main energy source for the body. The role of carbohydrate in the diabetes meal plan remains controversial. Traditionally, high carbohydrate, low fat diets were associated with lower prevalence of obesity and type 2 diabetes in the US in the early 20th century (i.e., before the mandatory B-vitamins fortification), and high-carbohydrate and low-fat diets were used for treating type 2 diabetes [16,17]. High carbohydrate diets, although inducing hyperlipidemia [33,34], were still found to improve glucose tolerance in the late 1960s and the early 1970s [35] (i.e., more than 20 years after the implementation of mandatory fortification). However, recently, increasing studies have shown that high carbohydrate diets increase the risk for obesity and type 2 diabetes, and that low carbohydrate diets may be beneficial for preventing obesity and type 2 diabetes in the past decade [8-10,36] (i.e., about 20 years after update of the fortification standards). Low-carbohydrate diets became a major weight loss and health maintenance trend in the US during the late 1990s and early 2000s [9,37]. The present study also revealed that the abrupt increase in prevalence of adult obesity was in parallel with the re-increase in the per capita carbohydrate consumption started from the early 1970s with a 10-year lag. It is assumed that the change in carbohydrate-diet effect may involve a change in consuming different type of carbohydrate [11]. Gross et al. suggested that the increased prevalence of diabetes in the US may be due to a high consumption of refined carbohydrates and a lack of fiber [8]. However, the fact is that there was a significant increasing trend in the per capita grain and fiber consumption (Figure 4E and Figure 7E, open cycles) and a decreasing trend in sugar contribution to per capita carbohydrate consumption (Figure 6D, open cycles) since about the early 1970s, which has been followed by a sharp increase, rather than by a decrease, in the prevalence of obesity and diabetes in the following three decades. Moreover, although it is suspected that increasing the consumption of fructose (mainly from beet or cane, high fructose corn syrup, fruits, and honey) may play a role in the prevalence of obesity and type 2 diabetes, there is, however, no unequivocal evidence that fructose intake at moderate doses is directly related with adverse metabolic effects [12]. Thus, it seems unlikely that the change in sugar consumption is responsible for the sharply increasing nationwide prevalence of obesity in the US started since the early 1980s.

Grain products, a major source of carbohydrate, are used as vehicles for the mandatory B-vitamins fortification. The mandatory grain fortification has led to a nationwide increase in B-vitamins intake [14]. Therefore, the adverse effects of B-vitamins fortification, if there are any, should be nationwide. Indeed, the present study found that the increased prevalence of adult obesity and diabetes in the US is highly correlated with the consumption of B-vitamin-fortified grains. Each of the two sharp increases in the vitamin contents, induced respectively by the initiation of the fortification and the update of the fortification standards, was followed by a nationwide increase in the prevalence of diabetes with a 26-year lag. More significantly, the update of grain fortification in 1974 and the subsequent increase use of fortified-grain products was followed by an abrupt increase in the prevalence of obesity among both the adults (Figure 3 and 5) and the children in the US with a 10-year lag [22].

Obesity is known to be associated with excessive energy intake. Indeed, the present population-based study also revealed a high correlation between the obesity prevalence and the per capita energy consumption (Figure 6F). Most of energy consumed by the US population is derived from grains, sugars, meat and fats/oils. The present data clearly showed that the contributions to per capita energy consumption from the known dietary risk factors for obesity and type 2 diabetes, such as meat and animal fats, are not increased or even decreased since the early 1970s. Therefore, it seems unlikely that these known dietary risk factors alone are responsible for the nationwide sharp increase in the prevalence of obesity since the late 1970s. An interesting finding from this analysis was the strong lag-correlation between high obesity prevalence and high fortified-grain contribution to the per capita energy consumption since the early 1970s, which is totally different from the association pattern of high unfortified-grain contribution to energy consumption with very low obesity prevalence in the early 20 century. Increase in fortified-grain contribution to the total energy consumption means an increase in the intake of fortified-grain and B-vitamins, which may lead to an excessive B-vitamin intake. Because B-vitamins can stimulate appetite [15], chronic excess B-vitamins may trigger excessive energy intake, which may contribute to the different outcomes of unfortified-grains and fortified-grains. This interpretation was further supported by the finding that the per capita B-vitamin consumption was lag-correlated not only with the per capita energy consumption but also with the prevalence of obesity and diabetes. Taken together, it seems quite possible that the nationwide increased prevalence of obesity and type 2 diabetes in the US in the late half of 20th century may involve an increase in B-vitamin consumption primarily due to the implementation of mandatory grain fortification with B-vitamins.

It should be noted that the standards of B-vitamins fortification vary from country to country in the world. For example, the level of wheat flour fortification with niacin in the US and the UK is 52.9 mg/kg and 16 mg/kg [13], respectively. Moreover, unlike in the US, the fortification in the UK is voluntary [13]. These differences may underlie regional differences in the study of carbohydrate effect. For example, even in the early 2000, a study from the UK still found that a low-fat, high-carbohydrate diet in overweight individuals with abnormal intermediary metabolism led to moderate weight loss and some improvement in serum cholesterol [38]. Thus, it seems necessary that the content of vitamins should be taken into consideration in the study of the relationship between carbohydrates and the development of obesity and diabetes.

### Excess niacin consumption and the obesity and diabetes prevalence

Niacin, one of the most stable of B vitamins, is resistant to heat, light, air, acid, and alkali [39], which means that, once added to grains, little is lost during food processing and cooking [40]. The well-known common adverse effects of niacin are metabolic disturbances, such as insulin resistance and glucose intolerance, and liver injury [15,19-22], all of which are the hallmarks of obesity and type 2 diabetes [2,4]. Our previous studies suggested that type 2 diabetes and obesity may involve excess niacin intake [21,22]. Although the prevalence of obesity and diabetes is also highly correlated with thiamin and riboflavin, however, so far as we know, there is no evidence yet indicating that either thiamin or riboflavin may induce glucose intolerance or insulin resistance [15]. Thus, it seems that the high prevalence of obesity and diabetes may involve niacin consumption.

Human dietary niacin comes mainly from two major sources: animal flesh (meat, poultry and fish) and grains, which accounts for about 70% of dietary niacin consumption in the US in the early 20th century [14]. The amount of the daily per capita niacin consumption from grains and animal flesh in the US was estimated to be 3.7 and 6.8 mg, respectively, in 1930s (just before the introduction of mandatory niacin-fortification), and has increased to 14.8 and 11.8 mg, respectively, in 2000, according the contribution of meat and grain to total niacin given in the literature [14,22]. The per capita niacin consumption from grains has increased four-fold since the implementation of niacin fortification. By the early 2000s, the US per capita daily niacin consumption has reached 33 mg [23], which is much higher than the RDA (see Introduction) [15]. Thus, long-term excess niacin intake may be very common in the US population after the implementation of mandatory niacin fortification, which may be mainly responsible for rapid increase in the prevalence of obesity and diabetes. According to the regression equations given in Figure 1B and Figure 3B, if the per capita niacin consumption is remained at the current levels (33 mg/d per capita), the prevalence of diabetes in the US would increase from the current about 6% to 7.6% by 2025, whereas the prevalence of obesity in the adults has reached its peak level. In agreement with this prediction, the recent NHANES data have shown that there was no significant change in the prevalence of obesity between 2003-2004 and 2005-2006 for either men or women [41].

### B-vitamins fortification and the global increasing obesity and diabetes prevalence

Grain fortification with B vitamins, a strategy for preventing B-vitamins deficiency, was first mandatorily implemented in the US in the early 1940s [42]. Soon after, many other industrialized countries, following the US model, have set up their own B-vitamins fortification programs [13,39]. During the last few decades, B-vitamins fortification of grains has also been introduced to developing countries [13]. Nowadays, vitamin fortification has become so popular in the world that, besides of grain fortification, other foods, such as most powdered milk and infant milk [43], have already been fortified with niacin and other B vitamins. Moreover, niacin has also been widely used in meat processing to maintain the bright red color of meat [44]. Although there are no data available concerning the relationships between B-vitamins fortification of a variety of foods and the global increasing prevalence of obesity and type 2 diabetes, the notable facts are that: (1) compared with formula-feeding, breastfeeding is associated with a reduction in risk of later overweight and obesity [45-47]; (2) high consumption of processed meat is a strong risk factor for type 2 diabetes [48]; and (3) the global prevalence of obesity and type 2 diabetes showed a trend of spreading from the earliest fortified-countries to the latter fortified-countries, whereas the non-vitamin-fortified countries including western developed countries, such as Norway, have a low prevalence of obesity and diabetes, compared with the earliest fortified-countries, such as the US and Canada [49,50]. The relevant evidence shows that, when the US was experiencing a rapid increase in the prevalence of diabetes in 1960s (i.e. more than 20 years after the introduction of B-vitamins fortification), the trend in the incidence of diabetes in Norwegian adults was fairly constant, or even significantly decreased in Norwegian women aged 40-59 years [51].

It has been well-recognized that physical inactivity also contributes substantially to the global epidemic of obesity and diabetes. A large scale investigation among the US physicians found that sweat-inducing exercise once a week may effectively reduce the risk of diabetes [52]. Although the exact mechanism of sweat-inducing physical activity is unclear, a well-known fact is that water-soluble B vitamins can be eliminated through sweat [53,54]. Our recent study also demonstrated that sauna-induced sweating may also effectively eliminate excess nicotinamide from the body and thus reduce the generation of toxic metabolites of nicotinamide [21]. In contrast, excess nicotinamide cannot be effectively eliminated through urine because of its reabsorption by the renal tubules [55]. Therefore, sweating is expected to play an important role in eliminating excess B-vitamins from the body. Unfortunately, modern lifestyle makes the sweat gland less active due to low physical activity and an increase in time spent in air-conditioned environments. The combination of high B-vitamins intake and low sweat elimination of excess B-vitamins may lead to chronic B-vitamins overload. Thus, there is a strong possibility that the worldwide spread of B-vitamins fortification of foods may play a role in the global prevalence of obesity and type 2 diabetes.

### The limitations of the current study

Diagnostic criteria are important for estimating the prevalence of diabetes. Because the NHIS survey was redesigned, two changes may have affected trends. First, the diabetes question was changed. Second, proxy respondents (i.e., household members responding for absent adult members) who tend to under report disease were no longer used in the survey [26,27]. The NHIS survey was redesigned in 1997, and since then gestational diabetes has been excluded. All diagnosed cases of diabetes in the US consist of type 2 diabetes (90% to 95%), type 1 diabetes (5% to 10%), gestational diabetes (2% to 5%) and other specific types of diabetes (1% to 2%) [56]. In this case, the prevalence of diabetes estimated should be lower after 1997 than before 1997 because of the exclusion of gestational diabetes. However, the fact is that the prevalence of diabetes has shown a steadily increasing trend since the late 1990s, and is significantly correlated with the per capita niacin consumption either before or after 1997 (Figure 1A). Moreover, before the significant increase in diabetes prevalence started from the mid-1990s, there had been a sudden increase in the prevalence of obesity in mid-1980s (Figure 3A). The diabetes prevalence is strongly correlated with the obesity prevalence with a time lag of 16 years. Considering that obesity is a major risk factor for type 2 diabetes, it is unlikely that the correlation between the B-vitamins consumption and the prevalence of diabetes is due to a sampling bias.

Ecological studies investigate relationships at the level of the group, rather than at the level of the individual. The inherent limitation of ecological studies is the ecological fallacy, i.e., the data of exposure and disease obtained from populations cannot be linked to individuals. To overcome this shortcoming, we used country-level food and nutrient disappearance data only from within the US, thus, the bias, if any, would at least be uniform for the same population. Also, we addressed the issue from different angles, by which the potential biases due to age or sex had been excluded. More distinctly, although this study is an ecological one, the exposure factor (the B-vitamins consumption) analysed in this study involves every adult inhabitant of the US due to the mandatory grain fortification. Moreover, the US standard of flour fortification with niacin is 5.29 mg/100 g, similar to the niacin content in meat, one of the richest sources of niacin (around 3.6 to 8.2 mg/100 g, depending on the particular type of meat product) [57]. In this case, no matter what dietary pattern is chosen, either high grain diet or high meat diet, the niacin exposure is essentially similar after the implementation of mandatory fortification.

It is important to examine evidence from a variety of sources and to look for congruence between epidemiologic, clinical and laboratory research findings before establishing causality between a diet factor and a human disease [58]. Although the present study provided only correlative evidence linked the B-vitamins consumption to the prevalence of obesity and type 2 diabetes, the following clinical and laboratory research findings provide support for a likely causal relationship between the high level of consumption of the B vitamins and the development of obesity and diabetes: (1) High grain intake-induced increase in the risk of obesity and type 2 diabetes in the US has occurred only after the implementation of B-vitamins fortification, especially after the update of the fortification standards in 1974. However, traditionally, low-fat, high-carbohydrate diets were beneficial for treatment of diabetes. (2) High intake of meat, a niacin-rich food, increases the risk for diabetes [7,48]. (3) Obesity is a well-known risk factor of type 2 diabetes, and the prevalence of obesity precedes the prevalence of diabetes. The present results showed that the time lag for the prevalence of obesity was much shorter than that for the prevalence of diabetes under the same exposure to the B-vitamins. (4) Niacin is well known to induce glucose intolerance and insulin resistance which are the key features of obesity and type 2 diabetes. (5) The prevalence of obesity and type 2 diabetes has spread in a way similar to that of B-vitamins fortification spread in the world, i.e., from developed countries to developing countries, but the non-vitamin-fortified countries including the developed countries have been less affected. (6) High-niacin feeding has been demonstrated to induce fatty liver in rats [59], and excess niacin intake may be involved in the development of nonalcoholic fatty liver disease, a disease closely associated obesity and type 2 diabetes [60]. (7) Niacin is a potent stimulator of appetite and may play a role in the development of obesity [22]. From the findings of this study, it seems that prospective studies are needed to evaluate the possible role of the high-level consumption of niacin, thiamin and riboflavin in the prevalence of obesity and type 2 diabetes.

## Conclusions

The present study revealed that the increased prevalence of obesity and diabetes in the US in the past 50 years was closely correlated with the increased daily per capita consumption of niacin, thiamin and riboflavin of with distinct time lags, and suggested that long-term exposure to high level of the B vitamins may be involved in the increasing prevalence of obesity and diabetes. The present findings, together with the evidence that niacin may induce glucose intolerance, insulin resistance and liver injury, imply the possibility that, among the fortified B-vitamins, excess niacin consumption may play a major role in the development of obesity and type 2 diabetes. Since the high level consumption of niacin in the US is mainly due to the implementation of mandatory grain fortification, therefore, it may be of significance to carefully evaluate the long-term safety of food fortification.

## Competing interests

The authors declare that they have no competing interests.

## Authors' contributions

SSZ contributed to the study conception and design and the data analysis and interpretation. DL, YMZ and WPS contributed to the collection and analysis of the data and drafting the manuscript. QGL participated in the design of the study and performed the statistical analysis. All authors read and approved the final manuscript.

## Pre-publication history

The pre-publication history for this paper can be accessed here:

http://www.biomedcentral.com/1471-2458/10/746/prepub
